# Damage Evolution and Life Prediction of Cross-Ply C/SiC Ceramic-Matrix Composite under Cyclic Fatigue Loading at Room Temperature and 800 °C in Air

**DOI:** 10.3390/ma8125474

**Published:** 2015-12-09

**Authors:** Longbiao Li

**Affiliations:** College of Civil Aviation, Nanjing University of Aeronautics and Astronautics, No. 29, Yudao St., Nanjing 210016, China; llb451@nuaa.edu.cn; Tel.: +86-25-8489-5963

**Keywords:** ceramic-matrix composites (CMCs), fatigue, damage evolution, life prediction, S-N curve

## Abstract

The damage evolution and life prediction of cross-ply C/SiC ceramic-matrix composite (CMC) under cyclic-fatigue loading at room temperature and 800 °C in air have been investigated using damage parameters derived from fatigue hysteresis loops, *i.e.*, fatigue hysteresis modulus and fatigue hysteresis loss energy. The experimental fatigue hysteresis modulus and fatigue hysteresis loss energy degrade with increasing applied cycles attributed to transverse cracks in the 90° plies, matrix cracks and fiber/matrix interface debonding in the 0° plies, interface wear at room temperature, and interface and carbon fibers oxidation at 800 °C in air. The relationships between fatigue hysteresis loops, fatigue hysteresis modulus and fatigue hysteresis loss energy have been established. Comparing experimental fatigue hysteresis loss energy with theoretical computational values, the fiber/matrix interface shear stress corresponding to different cycle numbers has been estimated. It was found that the degradation rate at 800 °C in air is much faster than that at room temperature due to serious oxidation in the pyrolytic carbon (PyC) interphase and carbon fibers. Combining the fiber fracture model with the interface shear stress degradation model and the fibers strength degradation model, the fraction of broken fibers *versus* the cycle number can be determined for different fatigue peak stresses. The fatigue life S-N curves of cross-ply C/SiC composite at room temperature and 800 °C in air have been predicted.

## 1. Introduction

Ceramic materials possess high strength and modulus at elevated temperatures. However, their use as structural components is severely limited because of their brittleness. Continuous fiber-reinforced ceramic-matrix composites (CMCs), by incorporating fibers in ceramic matrices, however, not only exploit their attractive high-temperature strength but also reduce the propensity for catastrophic failure. Carbon fiber-reinforced silicon carbide ceramic-matrix composites (C/SiC CMCs) are one of the most promising candidates for many high temperature applications, particularly as aerospace and aircraft thermostructural components [1]. The CMC flaps for exhaust nozzles of SNECMA M53 and M88 aero-engines have been used for more than one decade [2]. The CMC turbine vanes have been designed and tested in the aero-engine environment under the implementation of the Ultra Efficient Engine Technology (UEET) program [3]. A CMC turbine blade has been tested for 4 h by General Electric in a modified GE F414 engine, which represents the first application of CMC material in a rotating engine part. Incorporating the CMC turbine blades on a GE90-sized engine, the overall weight can be reduced by 455 kg, which represents ~6% of the dry weight of the full-sized GE90-115 [4]. The CMC combustion chamber floating wall tiles have also been tested in the aero-engine environment for 30 min, with a temperature range of 1047–1227 °C and a pressure of 2 MPa [5].

**Figure 1:**
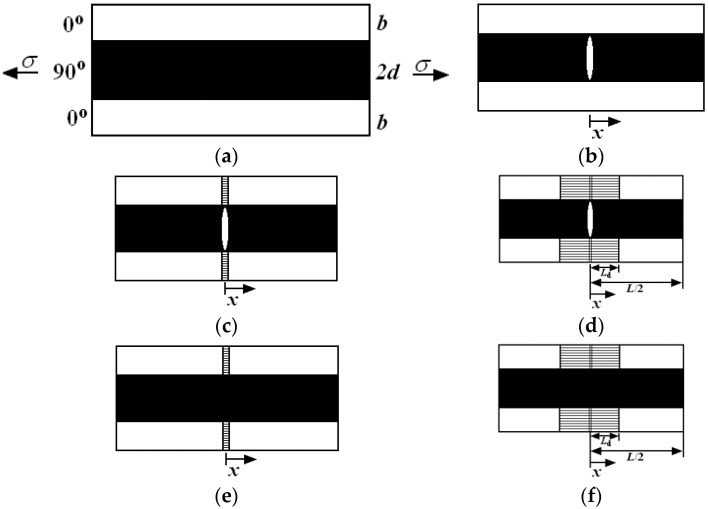
The undamaged state and five damaged modes of cross-ply ceramic composites: (**a**) undamaged composite; (**b**) mode 1: transverse cracking; (**c**) mode 2: transverse cracking and matrix cracking with perfect fiber/matrix bonding; (**d**) mode 3: transverse cracking and matrix cracking with fiber/matrix interface debonding; (**e**) mode 4: matrix cracking with perfect fiber/matrix bonding; and (**f**) mode 5: matrix cracking with fiber/matrix debonding.

CMCs are subject to fatigue upon cyclic mechanical and thermal loading. Understanding the damage mechanisms of fatigue represents an important step in engineering applications of these materials [6]. Zhu, *et al.* [7] investigated the tension-tension fatigue behavior of two-dimensional (2D) SiC/SiC composite at room temperature and 1000 °C in argon. It was found that the fatigue limit stress at 1000 °C in argon is much lower than that at room temperature. Mall and Engesser [8] investigated the effect of loading frequency on the tension-tension fatigue behavior of 2D C/SiC composite at 550 °C in air. It was found that there was no reduction in cycles to failure at 550 °C in air compared with that at room temperature at a high loading frequency of 375 Hz. Ruggles-Wrenn, *et al.* [9] investigated the effect of loading frequency and environment on the tension-tension fatigue behavior of 2D SiC/SiC composite at 1200 °C. It was found that the presence of steam significantly degraded the fatigue performance, and the fatigue lifetime decreased with the increasing loading frequency. Under cyclic-fatigue loading, the stress-strain hysteresis loop is an effective tool to indicate fatigue damage mechanisms of CMCs through analyzing the fatigue hysteresis modulus or fatigue hysteresis loops area [6,10,11,12]. For cross-ply CMCs, multiple matrix cracking modes affect the shape, location and area of hysteresis loops, which can be divided into five different types [13], *i.e.*, mode 1: transverse cracking; mode 2: transverse cracking and matrix cracking with perfect fiber/matrix interface bonding; mode 3: transverse cracking and matrix cracking with fiber/matrix interface debonding; mode 4: matrix cracking with perfect fiber/matrix interface bonding; and mode 5: matrix cracking with fiber/matrix interface debonding, as shown in Figure 1. Li, *et al.* [14,15] investigated the effect of matrix cracking modes on cyclic loading/unloading/reloading tensile hysteresis loops of cross-ply C/SiC composite. It was found that hysteresis loops can be used to monitor damage evolution in cross-ply CMCs under cyclic loading/unloading tensile, *i.e.*, the proportion of matrix cracking mode 3 in all the cracking modes of the composite can be determined through analyzing the hysteresis loops along with experimental observation under optical microscope.

The objective of this paper is to investigate the damage evolution and life prediction of cross-ply C/SiC composite under tension-tension cyclic-fatigue loading at room temperature and 800 °C in air using damage parameters derived from fatigue hysteresis loops, *i.e.*, fatigue hysteresis modulus and fatigue hysteresis loss energy. The experimental fatigue hysteresis modulus and fatigue hysteresis loss energy *versus* the cycle number curves have been analyzed. Comparing experimental fatigue hysteresis loss energy with theoretical computational values, the fiber/matrix interface shear stress corresponding to different cycle numbers has been estimated. Combining the fiber fracture model with the fiber/matrix interface shear stress degradation model and the fiber strength degradation model, the fraction of broken fibers *versus* the cycle number can be determined. The composite will fatigue-fracture when the fraction of broken fibers approaches the critical value. The fatigue life S-N curves of cross-ply C/SiC composite at room temperature and 800 °C in air have been predicted.

## 2. Material and Experimental Procedures

The T-700^TM^ carbon (Toray Institute Inc., Tokyo, Japan) fiber-reinforced silicon carbide matrix composites (C/SiC CMCs) were provided by Shanghai Institute of Ceramics, Shanghai, China. The fibers have an average diameter of 7 μm and come on a spool as a tow of 12 k fibers. The cross-ply C/SiC composite was manufactured by hot-pressing method, which offered the ability to fabricate dense composite via a liquid phase sintering method at a low temperature. The volume fraction of fibers was ~40%. The void content in the manufactured plates is below 5%. Low pressure chemical vapor infiltration was employed to deposit approximately 5–20 layer PyC/SiC with mean thickness of 0.2 μm in order to enhance the desired non-linear/non-catastrophic tensile behavior.

The dog bone-shaped specimens, with dimensions of 123 mm length, 3.8 mm thickness according to ASTM standard C 1360, and 10 mm width in the gauge section of cross-ply C/SiC composite, were cut from 150 mm × 150 mm panels by water cutting. The specimens were further coated with SiC of ~20 μm thick by chemical vapor deposition to prevent oxidation at elevated temperature.

The tension-tension fatigue experiments at room temperature and 800 °C in air were conducted on a MTS Model 809 servo hydraulic load-frame (MTS Systems Corp., Minneapolis, MN, USA) equipped with edge-loaded grips. The fatigue experiments were in a sinusoidal wave form and a loading frequency of 10 Hz without considering the visco-elastcity of carbon fibers [16,17]. The fatigue load ratio was 0.1, and the maximum number of applied cycles was defined to be 1,000,000 cycles. The fatigue tests were conducted under load control in accordance with the procedure in ASTM standard C 1360 at room temperature and 800 °C in air. The Multiple Purpose Testware (MPT) was used to program tension-tension fatigue tests and to acquire data throughout the duration of fatigue tests. After the start of fatigue testing, there needs to be a transition process to go through for the hydraulic machine to let the specimen’s actual stress amplitudes achieve desired levels. The length of transition process is relevant with the setting of Proportional Integral Derivative (PID) parameters [18]. However, the PID parameters are difficult to set for tension-tension fatigue tests of cross-ply C/SiC composite, due to their sensitivity to materials and test environments. For tension-tension fatigue tests conducted at room temperature, there needs a period of approximately 1000 cycles for the specimen to achieve the desired peak and valley stresses.

The stress levels in tension-tension fatigue tests were 90%, 85%, 80% and 70% of tensile strength at room temperature, and 70%, 65% and 60% of tensile strength at 800 °C in air. Under cyclic-fatigue loading, the displacement, strain and load were all recorded for each cycle. The fatigue hysteresis modulus *E* is calculated by [6],

(1) $$E=\frac{\sigma_{\max}-\sigma_{\min}}{\varepsilon_{\max}-\varepsilon_{\min}}$$

in which σ_max_ and σ_min_ denote the fatigue peak and valley stress, respectively; and ε_max_ and ε_min_ denote the fatigue peak and valley strain, respectively.

The area associated with fatigue hysteresis loops is the energy lost during corresponding cycles, which is defined as:

(2) $$U=\int_{\sigma_{\min}}^{\sigma_{\max}}\left[\varepsilon_{\text{c\_unload}}\left(\sigma \right)-\varepsilon_{\text{c\_reload}}\left(\sigma \right)\right]d\sigma$$

in which ε_c_unload_ and ε_c_reload_ denote the unloading and reloading composite strain, respectively.

The direct observation of matrix cracking under cyclic-fatigue tests was made by HiROX optical microscope (HiROX Co Ltd., Tokyo, Japan) at room temperature. The matrix crack density was determined by counting the number of cracks in a length of approximately 15 mm. For fatigue experiments at elevated temperature, the matrix crack density was observed after specimen fatigue failure. The fracture surfaces were also observed under optical microscope.

## 3. Experimental Results

### 3.1. Room Temperature

**Figure 2:**
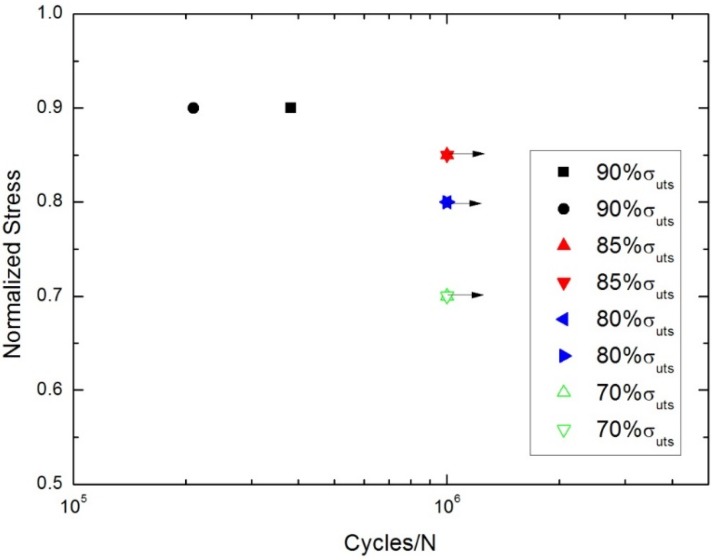
The tension-tension fatigue life S-N curve of cross-ply C/SiC composite at room temperature.

At room temperature, the tension-tension fatigue peak stresses were 112 MPa (90% tensile strength), 105 MPa (85% tensile strength), 99.2 MPa (80% tensile strength) and 87 MPa (70% tensile strength), respectively. The fatigue life S-N curve is shown in Figure 2.

**Figure 3:**
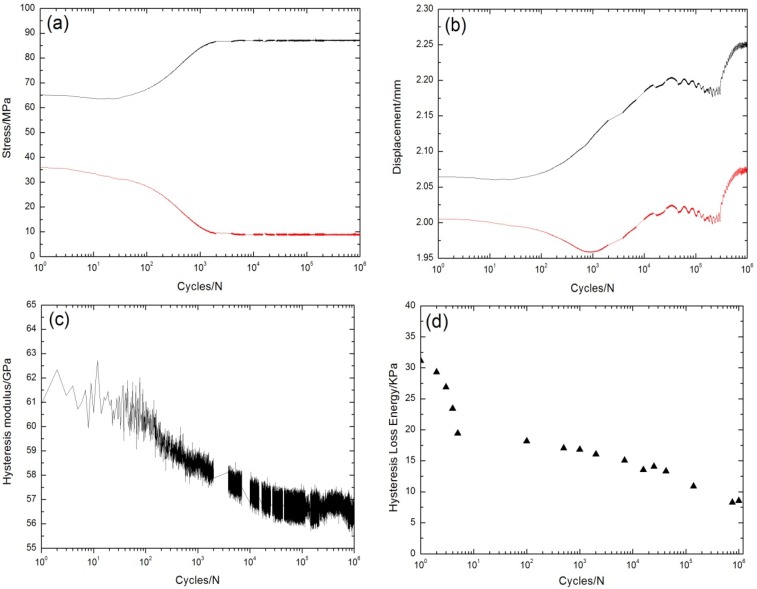
(**a**) The peak and valley stresses *versus* cycle number; (**b**) the peak and valley displacements *versus* cycle number; (**c**) the fatigue hysteresis modulus *versus* cycle number; and (**d**) the fatigue hysteresis loss energy *versus* cycle number of cross-ply C/SiC composite under σ_max_ = 87 MPa at room temperature.

Under σ_max_ = 87 MPa, the peak and valley stress *versus* cycle number curves are shown in Figure 3a. The displacements corresponding to the fatigue peak and valley stress increase with increasing applied cycles, as shown in Figure 3b. The fatigue hysteresis modulus decreases from 62 GPa at the first cycle to 56 GPa at the one-millionth cycle, as shown in Figure 3c. At the initial stage of cyclic-fatigue loading, the fatigue hysteresis modulus decreases due to transverse cracks in the 90° plies, and matrix cracks and fiber/matrix interface debonding in the 0° plies. With the increasing cycle number, the fatigue hysteresis modulus decreases slowly due to interface wear caused by the repeated frictional slip between fibers and the matrix [7,19,20]. The experimental fatigue hysteresis loss energy *versus* cycle number curve is shown in Figure 3d. The fatigue hysteresis loss energy degrades from 31.2 kPa at the first cycle to 8.5 kPa at the 996,403th cycle, attributed to the degradation of the fiber/matrix interface shear stress in the 0° plies caused by interface wear [11,12].

**Figure 4:**
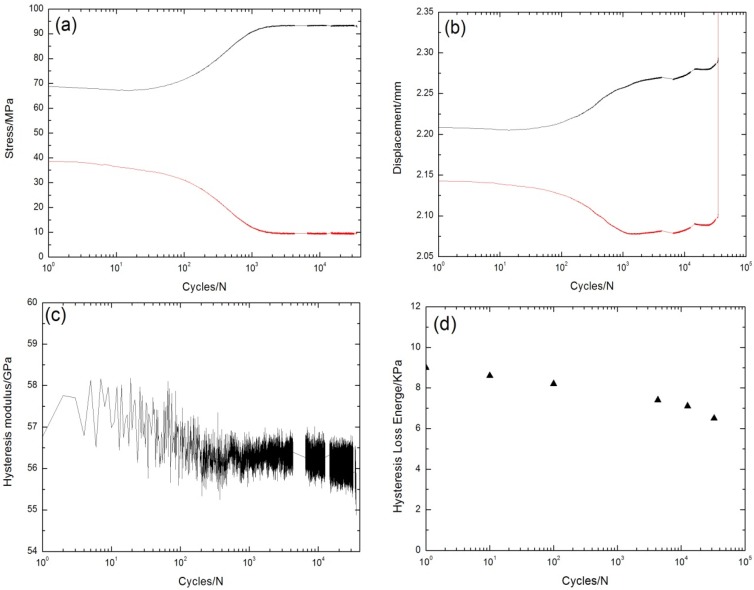
(**a**) The peak and valley stresses *versus* cycle number; (**b**) the peak and valley displacements *versus* cycle number; (**c**) the fatigue hysteresis modulus *versus* cycle number; and (**d**) the fatigue hysteresis loss energy *versus* cycle number of cross-ply C/SiC composite under σ_max_ = 93 MPa at room temperature.

The specimen without fatigue failure continued cyclic-fatigue loading at σ_max_ = 93 MPa. After experiencing 25,237 cycles, the specimen fatigue fractured. The fatigue peak and valley stresses *versus* cycle number curves are shown in Figure 4a. The displacements corresponding to the fatigue peak and valley stresses increase with the increasing cycle number, as shown in Figure 4b. The fatigue hysteresis modulus decreases from 56 GPa at the first cycle to 55 GPa at the 35,304th cycle, as shown in Figure 4c, due to new matrix cracks appearing between the original cracks at the low fatigue peak stress of σ_max_ = 87 MPa. The experimental fatigue hysteresis loss energy *versus* cycle number curve is shown in Figure 4d. The fatigue hysteresis loss energy degrades from 9 kPa at the first cycle to 6.5 kPa at the 32,079th cycle, due to the degradation of the fiber/matrix interface shear stress in the 0° plies [11,12].

**Figure 5:**
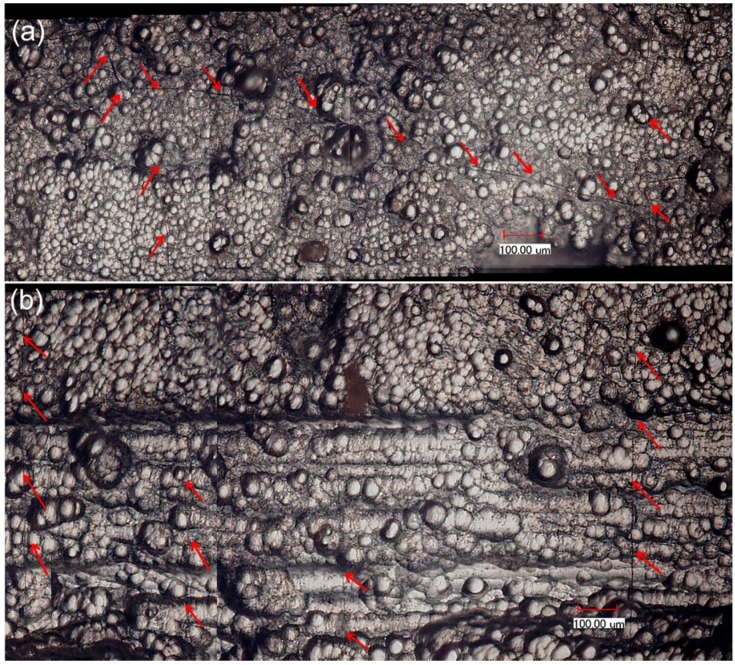

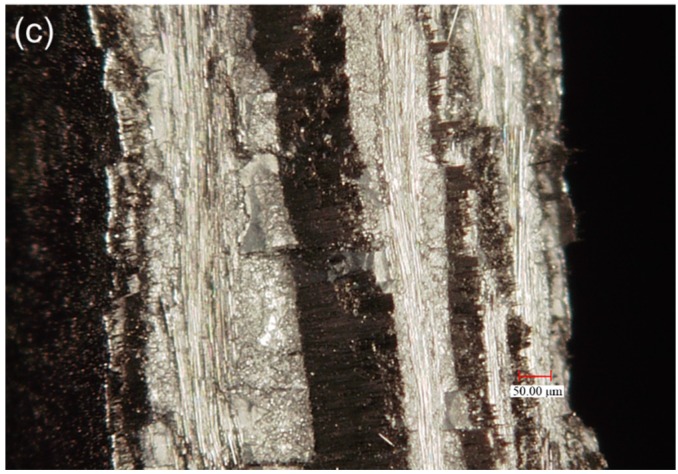
(**a**) The transverse cracks in the 90° plies; (**b**) the mode 3 and mode 5 cracks in 0° plies; and (**c**) the fracture surface of fatigue failure specimen at room temperature observed under optical microscope.

After the specimen fatigue failure, the side and fracture surfaces were observed under an optical microscope. The longitudinal cracks connect with the transverse cracks in the 90° plies, as shown in Figure 5a. There exist multiple mode 5 cracks and mode 3 major cracks, which propagate through the 90° and 0° plies. The proportion of mode 3 major cracks in all the matrix cracking modes of cross-ply composite is approximately 40%–45%, as shown in Figure 5b. There exist multiple fiber pullouts in the 0° plies, as shown in Figure 5c, which are mainly attributed to low interface shear stress caused by interface wear.

### 3.2. Elevated Temperature

**Figure 6:**
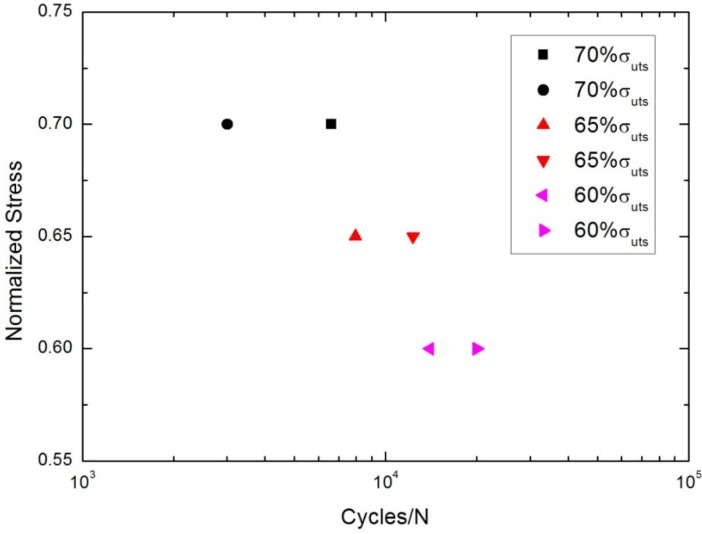
The tension-tension fatigue life S-N curve of cross-ply C/SiC composite at 800 °C in air.

At 800 °C in air, the tension-tension fatigue peak stresses were 105 MPa (70% tensile strength), 97.5 MPa (65% tensile strength) and 90 MPa (60% tensile strength), respectively. The fatigue life S-N curve is shown in Figure 6.

**Figure 7:**
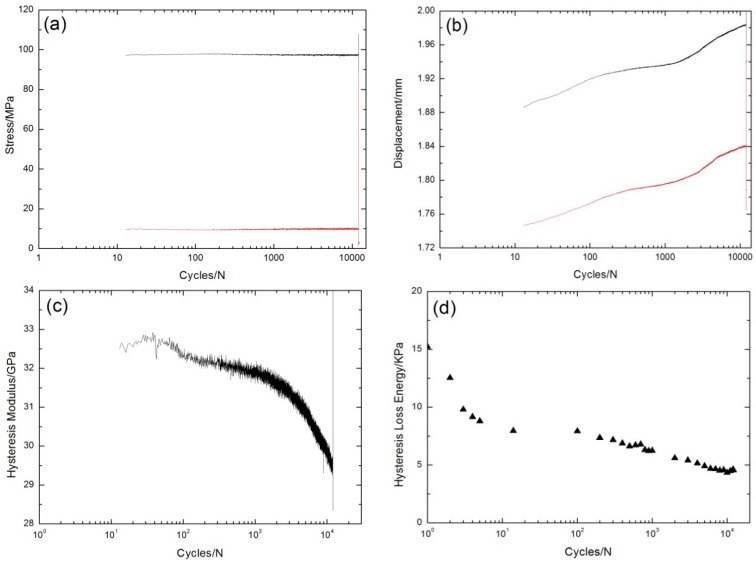
(**a**) The peak and valley stresses *versus* cycle number; (**b**) the peak and valley displacements *versus* cycle number; (**c**) the fatigue hysteresis modulus *versus* cycle number; and (**d**) the fatigue hysteresis loss energy *versus* cycle number of cross-ply C/SiC composite under σ_max_ = 97.5 MPa at 800 °C in air.

Under σ_max_ = 97.5 MPa, the peak and valley stress *versus* cycle number curves are shown in Figure 7a. The displacements corresponding to the fatigue peak and valley stress increase with the increasing cycle number, as shown in Figure 7b. The fatigue hysteresis modulus decreases gradually from 32.5 GPa at the 13th cycle to 31.8 GPa at the 1200th cycle, due to transverse cracks in the 90° plies, and matrix cracks and fiber/matrix interface debonding in the 0° plies; it then decreases rapidly to 28.6 GPa at the 12,096th cycle, which is attributed to interface oxidation and fiber fracture, as shown in Figure 7c. The experimental fatigue hysteresis loss energy *versus* cycle number curve is shown in Figure 7d. The fatigue hysteresis loss energy degrades from 15.2 kPa at the first cycle to 4.2 kPa at the 12,000th cycle, due to the degradation of interface shear stress caused by interface oxidation.

**Figure 8:**
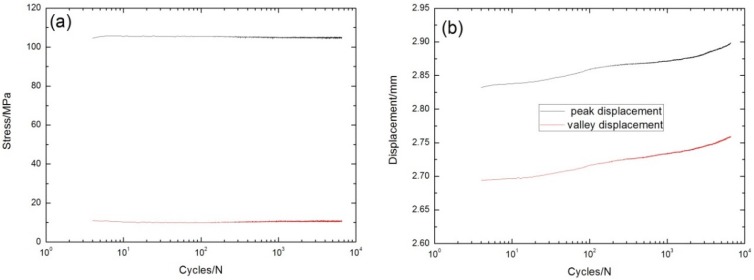

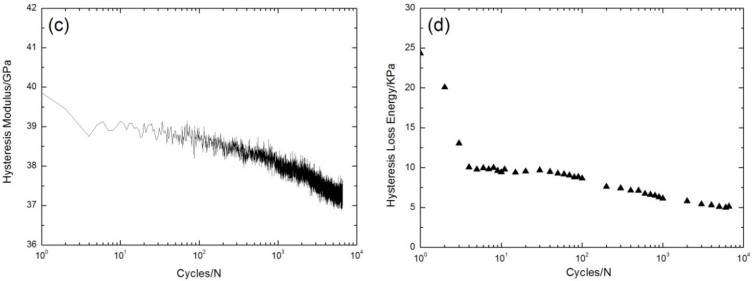
(**a**) The peak and valley stresses *versus* cycle number; (**b**) the peak and valley displacements *versus* cycle number; (**c**) the fatigue hysteresis modulus *versus* cycle number; and (**d**) the fatigue hysteresis loss energy *versus* cycle number of cross-ply C/SiC composite under σ_max_ = 105 MPa at 800 °C in air.

Under σ_max_ = 105 MPa, the peak and valley stress *versus* cycle number curves are shown in Figure 8a. The displacements corresponding to the fatigue peak and valley stress increase with the increasing cycle number, as shown in Figure 8b. The fatigue hysteresis modulus *versus* cycle number curve is given in Figure 8c, which can be divided into three regions, *i.e.*, (1) at the initial stage of cyclic-fatigue loading, the fatigue hysteresis modulus decreases rapidly due to transverse cracks in the 90° plies, and matrix cracks and fiber/matrix interface debonding in the 0° plies; (2) when matrix cracks approach saturation, the fatigue hysteresis modulus decreases slowly due to degradation of the fiber/matrix interface shear stress for interface oxidation; and (3) at the final cyclic-fatigue loading, the fatigue hysteresis modulus decreases rapidly due to fiber fracture. The experimental fatigue hysteresis loss energy *versus* cycle number curve is shown in Figure 8d. The fatigue hysteresis loss energy degrades from 24.3 kPa at the first cycle to 5.1 kPa at the 6600th cycle, due to degradation of the fiber/matrix interface shear stress caused by interface oxidation.

**Figure 9:**
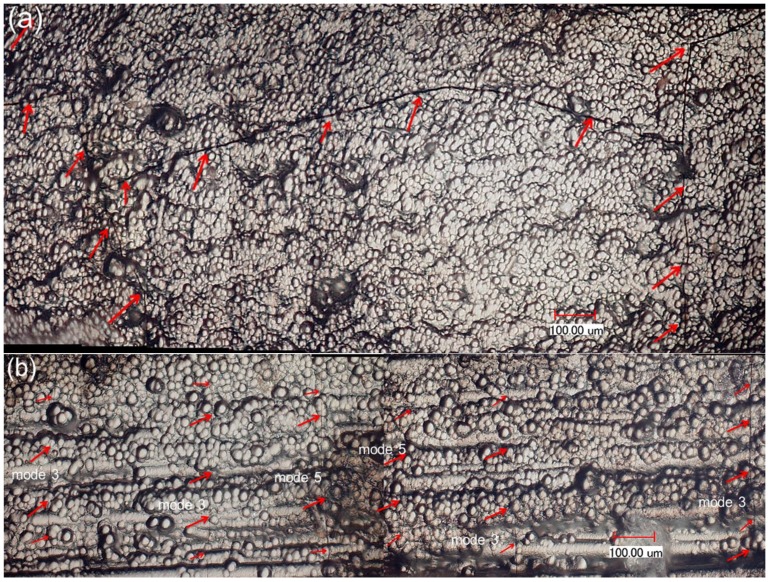

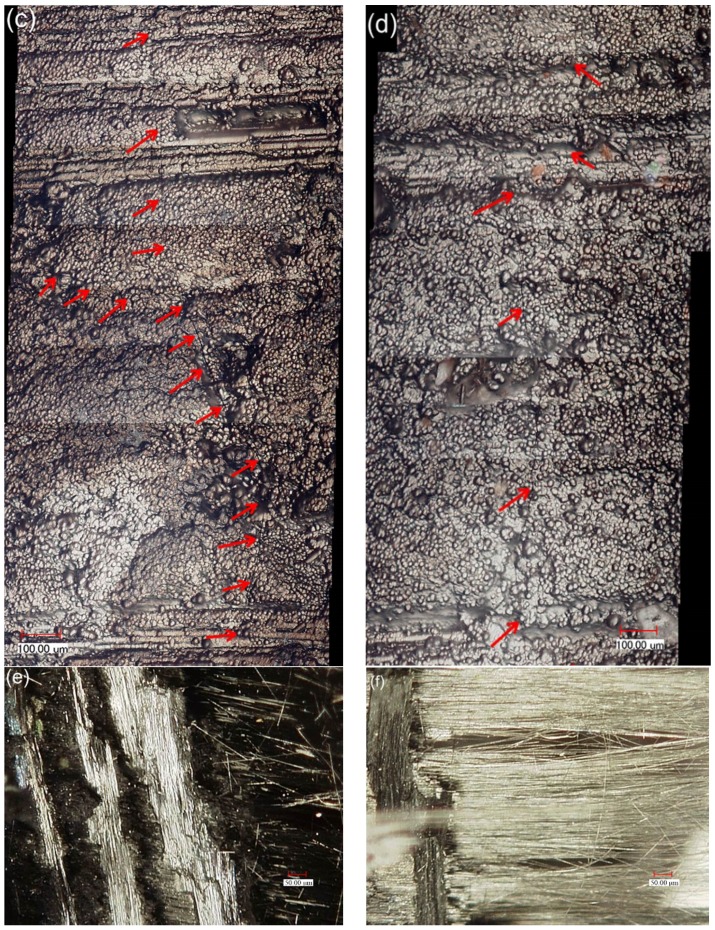
(**a**) The transverse cracks in the 90° plies; (**b**) the mode 3 and mode 5 cracks in the 0° plies; (**c**) the mode 3 cracks connect with transverse cracks; (**d**) the mode 3 cracks propagate through multiple 90° and 0° plies; (**e**) the pullout fibers at fracture surface; (**f**) the pullout fibers appear serious in oxidation of tension-tension fatigue failure specimen of cross-ply C/SiC composite at 800 °C in air observed under optical microscope.

After the specimen fatigue failure, the side and fracture surfaces were observed under an optical microscope. The longitudinal cracks connect with transverse cracks in the 90° plies, as shown in Figure 9a. In the 0° plies, there exist multiple mode 5 cracks and mode 3 major cracks, which propagate through the 90° and 0° plies. The proportion of mode 3 major cracks in all the matrix cracking modes of cross-ply composite is about 60%, as shown in Figure 9b. Some mode 3 major cracks propagate through multiple 90° and 0° plies, as shown in Figure 9c,d. There exist multiple fiber pullouts in 0° plies, as shown in Figure 9e,f, which are much longer than that of the fatigue failure specimen at room temperature, as shown in Figure 5c. The degradation of the interface shear stress caused by oxidation of PyC interphase and carbon fibers is the major reason for longer fiber pullout length under cyclic-fatigue loading at 800 °C in air.

## 4. Theoretical Analysis

### 4.1. Hysteresis Loops Model

If matrix multicracking and interface debonding are present upon the first cyclic-fatigue loading, the stress-strain hysteresis loops develop as a result of energy dissipation through frictional slip between fibers and the matrix. Based on the damage mechanism of frictional slip, the fatigue hysteresis loops of cross-ply CMCs can be divided into four cases, *i.e.*, Case 1: interface partially debonds and fiber slips completely relative to the matrix; Case 2: interface partially debonds and fiber slips partially relative to the matrix; Case 3: interface completely debonds and fiber slips partially relative to the matrix; and Case 4: interface completely debonds and fiber slips completely relative to the matrix in the interface debonded region of the 0° plies upon unloading/reloading. It is assumed that fatigue hysteresis loops of cross-ply CMCs develop mainly due to frictional slip in the interface debonded region of matrix cracking mode 3 and mode 5, as shown in Figure 1.

For matrix cracking mode 3, the unloading and reloading strains when the interface partially debonds are [14,15]:

(3a) $$\varepsilon_{c\_unload}=\frac{\sigma }{V_{f0}E_{f}}+4\frac{\tau_{i}}{E_{f}}\frac{y^{2}}{r_{f}l_{c}}-2\frac{\tau_{i}}{E_{f}}\frac{\left(2y-l_{d}\right)\left(2y-l_{c}+l_{d}\right)}{r_{f}l_{c}}-\left(\alpha_{c}-\alpha_{f}\right)\Delta Τ$$

(3b) $$\begin{array}{ll} \varepsilon_{c}=\frac{\sigma }{V_{f0}E_{f}} & -4\frac{\tau_{i}}{E_{f}}\frac{z^{2}}{r_{f}l_{c}}+\frac{4\tau_{i}}{E_{f}}\frac{{\left(y-2z\right)}^{2}}{r_{f}l_{c}}\\ & +2\frac{\tau_{i}}{E_{f}}\frac{\left(l_{d}-2y+2z\right)\left(l_{d}+2y-2z-l_{c}\right)}{r_{f}l_{c}}-\left(\alpha_{c}-\alpha_{f}\right)\Delta Τ \end{array}$$

in which *V*_f0_ denotes the fiber volume content in the 0° direction; *E*_f_ denotes the fiber elastic modulus; *r*_f_ denotes the fiber radius; τ_i_ denotes the interface shear stress in the 0° ply; *l*_c_ denotes the matrix crack spacing; *l*_d_ denotes the interface debonded length; α_f_ and α_c_ denote the fiber and composite thermal expansion coefficient, respectively; Δ*T* denotes the temperature difference between fabricated temperature *T*_0_ and room temperature *T*_1_ (Δ*T* = *T*_1_ − *T*_0_); and *y* and *z* denote the interface counter-slip length and interface new-slip length, respectively.

When the interface completely debonds, the unloading and reloading strains are [14,15]:

(4a) $$\varepsilon_{c\_unload}=\frac{\sigma }{V_{f0}E_{f}}+4\frac{\tau_{i}}{E_{f}}\frac{y^{2}}{r_{f}l_{c}}-2\frac{\tau_{i}}{E_{f}}\frac{{\left(2y-l_{c}/2\right)}^{2}}{r_{f}l_{c}}-\left(\alpha_{c}-\alpha_{f}\right)\Delta Τ$$

(4b) $$\varepsilon_{\text{c\_reload}}=\frac{\sigma }{V_{\text{f0}}E_{\text{f}}}-4\frac{\tau_{\text{i}}}{E_{\text{f}}}\frac{z^{2}}{r_{\text{f}}l_{\text{c}}}+4\frac{\tau_{\text{i}}}{E_{\text{f}}}\frac{{\left(y-2z\right)}^{2}}{r_{\text{f}}l_{\text{c}}}-2\frac{\tau_{\text{i}}}{E_{\text{f}}}\frac{{\left(l_{\text{c}}/2-2y+2z\right)}^{2}}{r_{\text{f}}l_{\text{c}}}-\left(\alpha_{\text{c}}-\alpha_{\text{f}}\right)\Delta Τ$$

For matrix cracking mode 5, the unloading and reloading strains when the interface partially debonds are [14,15]:

(5a) $$\varepsilon_{\text{c\_unload}}=\frac{1}{V_{\text{f0}}E_{\text{f}}}\left(\sigma -k\sigma_{\text{to}}\right)+4\frac{\tau_{\text{i}}}{E_{\text{f}}}\frac{y^{2}}{r_{\text{f}}l_{\text{c}}}-2\frac{\tau_{\text{i}}}{E_{\text{f}}}\frac{\left(2y-l_{\text{d}}\right)\left(2y+l_{\text{d}}-l_{\text{c}}\right)}{r_{\text{f}}l_{\text{c}}}-\left(\alpha_{\text{c}}-\alpha_{\text{f}}\right)\Delta Τ$$

(5b) $$\begin{array}{ll} \varepsilon_{\text{c\_reload}}= & \frac{1}{V_{\text{f0}}E_{\text{f}}}\left(\sigma -k\sigma_{\text{to}}\right)-4\frac{\tau_{\text{i}}}{E_{\text{f}}}\frac{z^{2}}{r_{\text{f}}l_{\text{c}}}+\frac{4\tau_{\text{i}}}{E_{\text{f}}}\frac{{\left(y-2z\right)}^{2}}{r_{\text{f}}l_{\text{c}}}\\ & \;+2\frac{\tau_{\text{i}}}{E_{\text{f}}}\frac{\left(l_{\text{d}}-2y+2z\right)\left(l_{\text{d}}+2y-2z-l_{\text{c}}\right)}{r_{\text{f}}l_{\text{c}}}-\left(\alpha_{c}-\alpha_{\text{f}}\right)\Delta Τ \end{array}$$

in which *k* denotes the proportion of 90° plies in the entire composite.

When the interface completely debonds, the unloading and reloading strains are [14,15]:

(6a) $$\varepsilon_{\text{c\_unload}}=\frac{1}{V_{\text{f0}}E_{\text{f}}}\left(\sigma -k\sigma_{\text{to}}\right)+4\frac{\tau_{\text{i}}}{E_{\text{f}}}\frac{y^{2}}{r_{\text{f}}l_{\text{c}}}-2\frac{\tau_{\text{i}}}{E_{\text{f}}}\frac{{\left(2y-l_{\text{c}}/2\right)}^{2}}{r_{\text{f}}l_{\text{c}}}-\left(\alpha_{\text{c}}-\alpha_{\text{f}}\right)\Delta Τ$$

(6b) $$\begin{array}{ll} \varepsilon_{\text{c\_reload}}= & \frac{1}{V_{\text{f0}}E_{\text{f}}}\left(\sigma -k\sigma_{\text{to}}\right)-4\frac{\tau_{\text{i}}}{E_{\text{f}}}\frac{z^{2}}{r_{\text{f}}l_{\text{c}}}+4\frac{\tau_{\text{i}}}{E_{\text{f}}}\frac{{\left(y-2z\right)}^{2}}{r_{\text{f}}l_{\text{c}}}\\ & -2\frac{\tau_{\text{i}}}{E_{\text{f}}}\frac{{\left(l_{\text{c}}/2-2y+2z\right)}^{2}}{r_{\text{f}}l_{\text{c}}}-\left(\alpha_{\text{c}}-\alpha_{\text{f}}\right)\Delta Τ \end{array}$$

Substituting unloading and reloading strains of Equations (3) and (4) into Equation (2), the fatigue hysteresis loss energy *U*_3_ of matrix cracking mode 3 can be obtained for different interface slip cases. Substituting unloading and reloading strains of Equations (5) and (6) into Equation (2), the fatigue hysteresis loss energy *U*_5_ of matrix cracking mode 5 can also be obtained for different interface slip cases. The fatigue hysteresis loss energy *U*_c_ of cross-ply CMCs is [14,15]:

(7) $$U_{\text{c}}=\eta U_{3}+\left(1-\eta \right)U_{5}$$

in which η denotes the composite damage parameter, *i.e.*, the proportion of matrix cracking mode 3 in all the cracking modes of the composite, and η ∈ [0,1].

### 4.2. Life Prediction Model

Under cyclic-fatigue loading at constant peak stress, fibers break due to degradation of the interface shear stress and fiber strength for interface wear [6,7,11] at room temperature or interface oxidation at elevated temperatures [8,12]. When fibers break, the load dropped by broken fibers would transfer to intact fibers at the cross-section. The Global Load Sharing criterion is adopted to determine the load carried by intact and fracture fibers [21].

(8) $$\frac{\sigma }{V_{\text{f0}}}=T\left[1-P\left(T\right)\right]+\langle T_{\text{b}}\rangle P\left(T\right)$$

in which <*T*_b_> denotes the load carried by broken fibers; and *P*(*T*) denotes the fraction of broken fibers.

(9) $$P\left(T\right)=1-\exp\left\{-{\left(\frac{T}{\sigma_{\text{c}}}\right)}^{m_{\text{f}}+1}{\left(\frac{\sigma_{\text{o}}}{\sigma_{\text{o}}\left(N\right)}\right)}^{m_{\text{f}}}\frac{\tau_{\text{i}}}{\tau_{\text{i}}\left(N\right)}\right\}$$

in which *m*_f_ denotes the fiber Weibull modulus; and σ_c_ denotes the fiber characteristic strength of a length δ_c_ [22].

(10) $$\sigma_{\text{c}}={\left(\frac{l_{\text{o}}\sigma_{\text{o}}^{m_{\text{f}}}\tau_{\text{i}}}{r_{\text{f}}}\right)}^{1/m_{\text{f}}+1},\delta_{\text{c}}={\left(\frac{\sigma_{\text{o}}r_{\text{f}}l_{\text{o}}^{1/m_{\text{f}}}}{\tau_{\text{i}}}\right)}^{m_{\text{f}}/m_{\text{f}}+1}$$

in which *l*_o_ is the reference length; and σ_o_ is the fiber reference strength of a length *l*_o_; σ_o_(*N*) is the fiber strength at the Nth cycle. Lee and Stinchcomb [23] conducted fiber fracture mirror experiments under scanning electron microscope (SEM), and found that fiber strength degraded with the increasing cycle number.

(11) $$\sigma_{\text{o}}\left(N\right)=\sigma_{\text{o}}\left[1-p_{1}{\left(\log N\right)}^{p_{2}}\right]$$

in which *p*_1_ and *p*_2_ are empirical parameters. τ_i_(*N*) is the interface shear stress at the Nth cycle. Evans, *et al.* [6] proposed the interface shear stress degradation model based on the analysis of fatigue hysteresis loops.

(12) $$\tau_{\text{i}}\left(N\right)=\tau_{\text{io}}+\left[1-\exp\left(-\omega N^{\lambda }\right)\right]\left(\tau_{\text{imin}}-\tau_{\text{io}}\right)$$

in which τ_io_ denotes the initial interface shear stress; τ_imin_ denotes the steady-state interface shear stress; and ω and λ are empirical parameters.

When fibers break, the fiber stress drops to zero at the break point. The stress in the fiber builds up through interface shear stress, as determined by Equation (13).

(13) $$T_{\text{b}}\left(x\right)=\frac{2\tau_{\text{i}}\left(N\right)}{r_{\text{f}}}x$$

The slip length *l*_f_ required to build the fiber stress up to its previous intact value is given by Equation (14).

(14) $$l_{\text{f}}=\frac{r_{\text{f}}T}{2\tau_{\text{i}}\left(N\right)}$$

The probability distribution *f*(*x*) of the distance *x* of a fiber break from the reference matrix cracking plane, provided that a break occurs within a distance ±*l*_f_, is determined by Equation (15) [22].

(15) $$\begin{array}{ll} f\left(x\right)= & \frac{1}{P\left(T\right)l_{\text{f}}}{\left(\frac{T}{\sigma_{\text{c}}}\right)}^{m_{\text{f}}+1}{\left(\frac{\sigma_{\text{o}}}{\sigma_{\text{o}}\left(N\right)}\right)}^{m_{\text{f}}}\frac{\tau_{\text{i}}}{\tau_{\text{i}}\left(N\right)}\\ & \times \exp\left\{-\left(\frac{x}{l_{\text{f}}}\right){\left(\frac{T}{\sigma_{\text{c}}}\right)}^{m_{\text{f}}+1}{\left(\frac{\sigma_{\text{o}}}{\sigma_{\text{o}}\left(N\right)}\right)}^{m_{\text{f}}}\frac{\tau_{\text{i}}}{\tau_{\text{i}}\left(N\right)}\right\},x\in \left[0,l_{\text{f}}\right] \end{array}$$

Using Equations (13)–(15), the average stress carried by broken fibers is determined by Equation (16).

(16) $$\begin{array}{l} \langle T_{\text{b}}\rangle =\int_{0}^{l_{\text{f}}}T_{\text{b}}\left(x\right)f\left(x\right)dx\\ =\frac{T}{P\left(T\right)}{\left(\frac{\sigma_{\text{c}}}{T}\right)}^{m_{\text{f}}+1}{\left(\frac{\sigma_{\text{o}}\left(N\right)}{\sigma_{\text{o}}}\right)}^{m_{\text{f}}}\frac{\tau_{\text{i}}\left(N\right)}{\tau_{\text{i}}}\left\{1-\exp\left[-{\left(\frac{T}{\sigma_{\text{c}}}\right)}^{m_{\text{f}}+1}{\left(\frac{\sigma_{\text{o}}}{\sigma_{\text{o}}\left(N\right)}\right)}^{m_{\text{f}}}\frac{\tau_{\text{i}}}{\tau_{\text{i}}\left(N\right)}\right]\right\}\\ \;\;\;\;-\frac{T}{P\left(T\right)}\exp\left\{-{\left(\frac{T}{\sigma_{\text{c}}}\right)}^{m_{\text{f}}+1}{\left(\frac{\sigma_{\text{o}}}{\sigma_{\text{o}}\left(N\right)}\right)}^{m_{\text{f}}}\frac{\tau_{\text{i}}}{\tau_{\text{i}}\left(N\right)}\right\} \end{array}$$

Substituting Equation (16) into Equation (8), it leads to the form of:

(17) $$\frac{\sigma }{V_{\text{f0}}}=T{\left(\frac{\sigma_{\text{c}}}{T}\right)}^{m_{\text{f}}+1}{\left(\frac{\sigma_{\text{o}}\left(N\right)}{\sigma_{\text{o}}}\right)}^{m_{\text{f}}}\frac{\tau_{\text{i}}\left(N\right)}{\tau_{\text{i}}}\left\{1-\exp\left[-{\left(\frac{T}{\sigma_{\text{c}}}\right)}^{m_{\text{f}}+1}{\left(\frac{\sigma_{\text{o}}}{\sigma_{\text{o}}\left(N\right)}\right)}^{m_{\text{f}}}\frac{\tau_{\text{i}}}{\tau_{\text{i}}\left(N\right)}\right]\right\}$$

Using Equations (11), (12) and (17), the stress *T* carried by intact fibers at the matrix cracking plane can be determined for different fatigue peak stresses. Substituting Equations (11) and (12) and the intact fiber stress *T* into Equation (9), the fiber fracture probability for different cycle numbers can be determined. When the fraction of broken fibers approaches the critical value q*, the composite would fatigue-fracture [21].

(18) $$q^{*}=\frac{2}{m_{\text{f}}+2}$$

**Figure 10:**
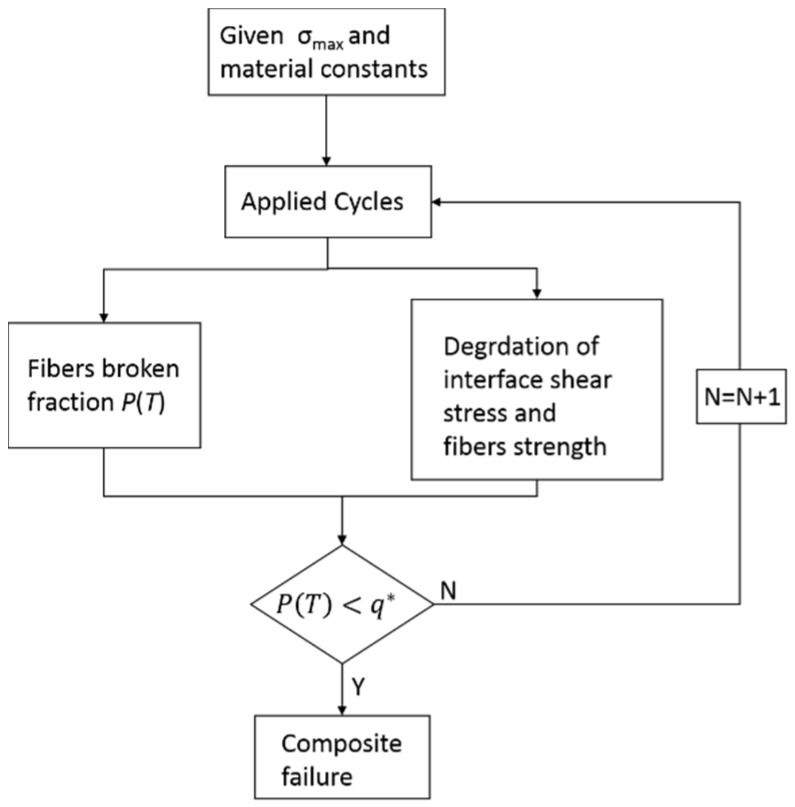
The schematic of the computational procedure for life prediction under cyclic-fatigue loading.

The flow chart of the life prediction model in the present analysis is given in Figure 10.

## 5. Experimental Comparisons

The basic material properties of cross-ply C/SiC composite are given by: *V*_f_ = 40%, *E*_f_ = 230 GPa, *E*_m_ = 350 GPa, *r*_f_ = 3.5 μm, α_f_ = −0.38 × 10^−6^/°C, α_m_ = 2.8 × 10^−6^/°C, Δ*T* = −1000 °C, σ_c_ = 4.9 GPa and *m*_f_ = 5. The interface shear stress corresponding to different cycle numbers is estimated by comparing the experimental fatigue hysteresis loss energy with theoretical computational values.

### 5.1. Room Temperature

**Figure 11:**
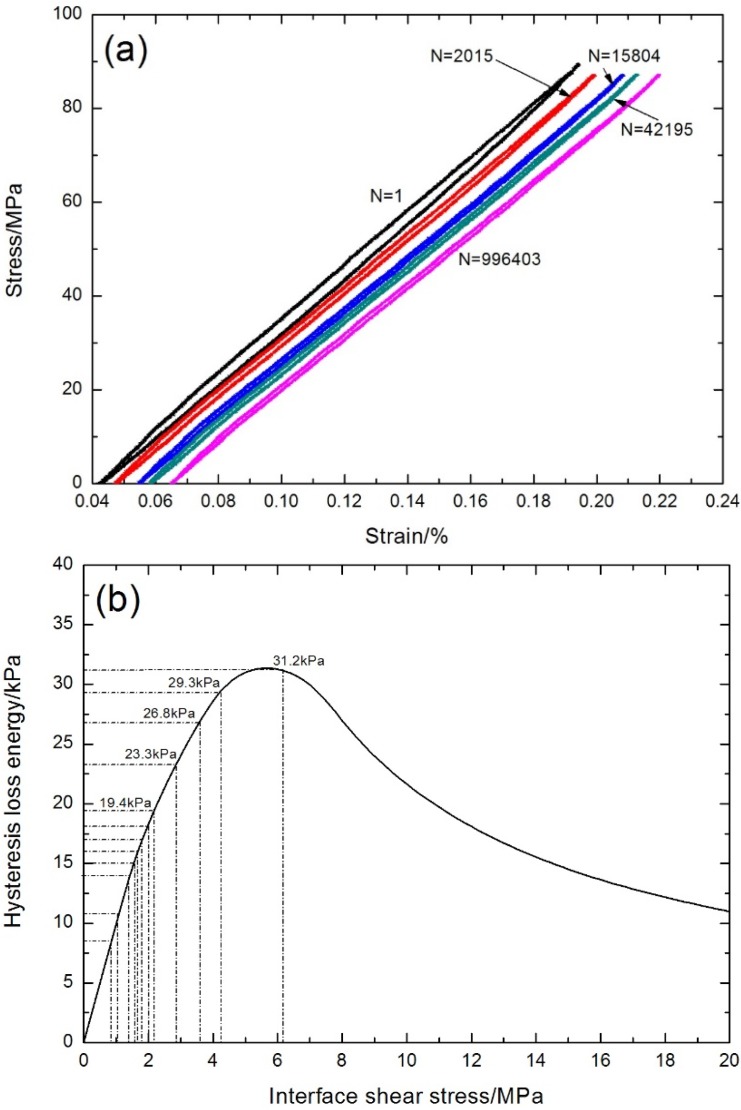
(**a**) The experimental fatigue hysteresis loops of different numbers of applied cycles; and (**b**) the theoretical fatigue hysteresis loss energy as a function of interface shear stress of cross-ply C/SiC composite under σ_max_ = 87 MPa at room temperature.

The fatigue hysteresis loops under σ_max_ = 87 MPa corresponding to the first, 2015th, 15,804th, 42,195th, and 996,403th cycles are shown in Figure 11a. The theoretical fatigue hysteresis loss energy as a function of interface shear stress is shown in Figure 11b. The fatigue hysteresis loss energy increases with decreasing interface shear stress to the peak value of 31.3 kPa, corresponding to interface slip Case 1 and Case 2, *i.e.*, interface partially debonding and fiber slipping completely/partially relative to the matrix in the interface debonded region of the 0° plies; it decreases with decreasing interface shear stress to 0 kPa, corresponding to interface slip Case 3 and Case 4, *i.e.*, interface completely debonding and fiber slipping partially/completely relative to the matrix in the interface debonded region of the 0° plies. Comparing the experimental fatigue hysteresis loss energy with theoretical computational values, the interface shear stress corresponding to the first, second, third, fourth, fifth, 100th, 500th, 2015th, 7026th, 25,098th, 139,744th, and 996,403th cycles can be estimated, as shown in Table 1.

**Table 1:** The fiber/matrix interface shear stress of cross-ply C/SiC composite corresponding to different numbers of applied cycles under σ_max_ = 87 MPa at room temperature.

Loading Cycles	Experimental Fatigue Hysteresis Loss Energy/kPa	Interface Shear Stress/MPa
1	31.2	6.2
2	29.3	4.3
3	26.8	3.6
4	23.3	2.8
5	19.4	2.2
100	18.1	2
500	17	1.8
2015	16	1.7
7026	15	1.5
25,098	14	1.4
139,744	10.8	1
996,403	8.5	0.85

**Figure 12:**
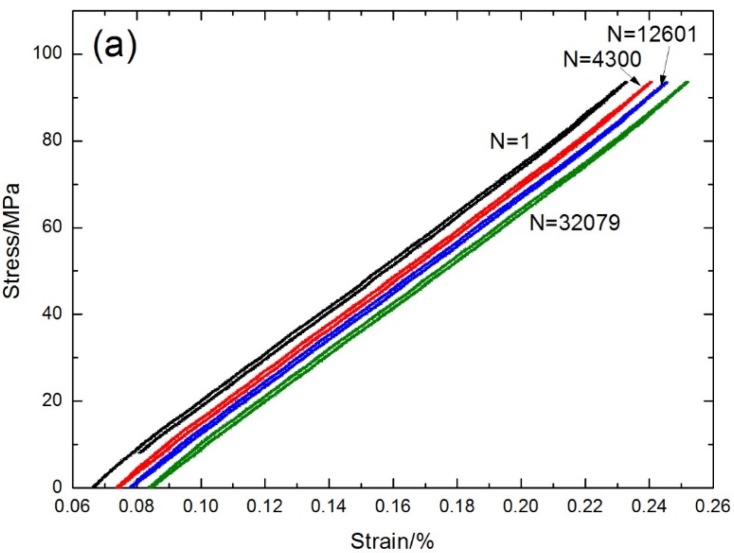

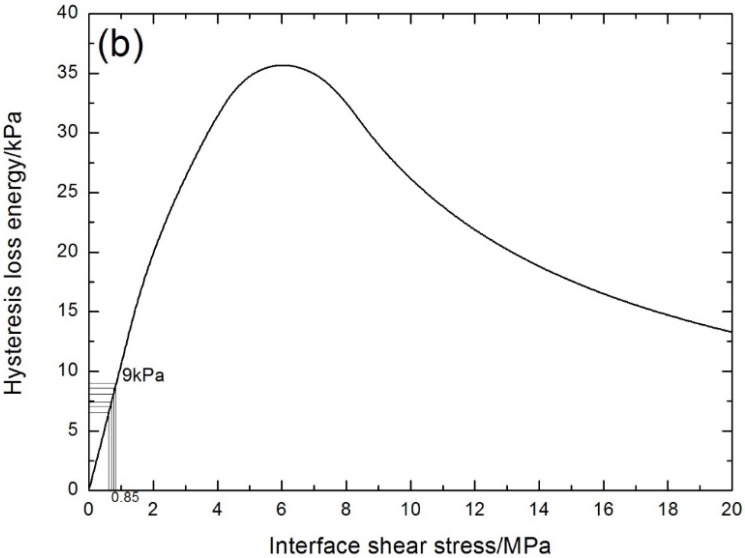
(**a**) The experimental fatigue hysteresis loops of different numbers of applied cycles; and (**b**) the theoretical fatigue hysteresis loss energy as a function of interface shear stress of cross-ply C/SiC composite under σ_max_ = 93 MPa at room temperature.

The fatigue hysteresis loops under σ_max_ = 93 MPa corresponding to the first, 4300th, 12,601th, and 32,079th cycles are shown in Figure 12a. The theoretical fatigue hysteresis loss energy as a function of interface shear stress is shown in Figure 12b. The fatigue hysteresis loss energy increases with decreasing interface shear stress to the peak value of 35.6 kPa, corresponding to interface slip Case 1 and Case 2, *i.e.*, interface partially debonding and fiber slipping completely/partially relative to the matrix in the interface debonded region of 0° plies; it decreases with decreasing interface shear stress to 0 kPa, corresponding to interface slip Case 3 and Case 4, *i.e.*, interface completely debonding and fiber slipping partially/completely relative to the matrix in the interface debonded region of 0° plies. Comparing experimental fatigue hysteresis loss energy with theoretical computational values, the interface shear stress corresponding to the first, 10th, 100th, 1000th, 4300th, 12,601th, and 32,079th cycles can be estimated, as shown in Table 2.

**Table 2:** The fiber/matrix interface shear stress of cross-ply C/SiC composite corresponding to different numbers of applied cycles under σ_max_ = 93 MPa at room temperature.

Loading Cycles	Experimental Fatigue Hysteresis Loss Energy/kPa	Interface Shear Stress/MPa
1	9	0.85
10	8.6	0.82
100	8.2	0.78
4300	7.4	0.7
12,601	7.1	0.68
32,079	6.5	0.62

**Figure 13:**
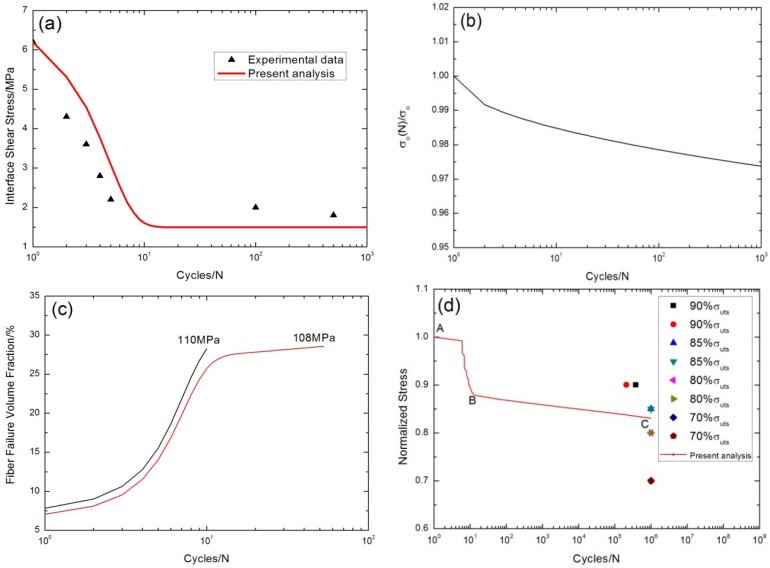
(**a**) The interface shear stress *versus* cycle number; (**b**) the fiber strength *versus* cycle number; (**c**) the fraction of broken fibers *versus* cycle number; and (**d**) the fatigue life S-N curves of experimental data and present analysis of cross-ply C/SiC composite at room temperature.

The interface shear stress as a function of the cycle number has been simulated using the Evans-Zok-McMeeking model [6], as shown in Figure 13a. The model parameters are given by τ_io_ = 6.2 MPa, τ_imin_ = 1.5 MPa, ω = 0.06 and λ = 1.8. The interface shear stress degrades from 6.2 MPa at the first cycle to 2 MPa at the 100th cycle due to interface wear, as shown in Figure 13a. The fiber strength degradation curve predicted using the Lee-Stinchcomb model [23] is shown in Figure 13b, in which *p*_1_ = 0.01 and *p*_2_ = 0.8. The fraction of broken fibers under σ_max_ = 110 MPa and 108 MPa corresponding to different cycle numbers predicted using the present analysis is illustrated in Figure 13c. Under σ_max_ = 108 MPa, the fraction of broken fibers *versus* cycle number curve can be divided into two regions, *i.e.*, (1) at the first 10 cycles, the fraction of broken fibers increases rapidly due to degradation of the interface shear stress and the fiber strength; (2) when interface shear stress approaches to steady-state value, the fiber fracture is mainly controlled by the degradation of fiber strength, which makes the fraction of broken fibers increase slowly, as shown in Figure 13c. The experimental and predicted fatigue life S-N curves of cross-ply C/SiC composite are illustrated in Figure 13d. The fatigue limit approaches 88% of the tensile strength. The predicted fatigue life S-N curve can be divided into two regions, *i.e.*, (1) the A–B part is affected by the degradation of the interface shear stress and the fiber strength for interface wear; and (2) the B–C part is mainly affected by the degradation of the fiber strength for interface wear, as shown in Figure 13d.

### 5.2. Elevated Temperature

**Figure 14:**
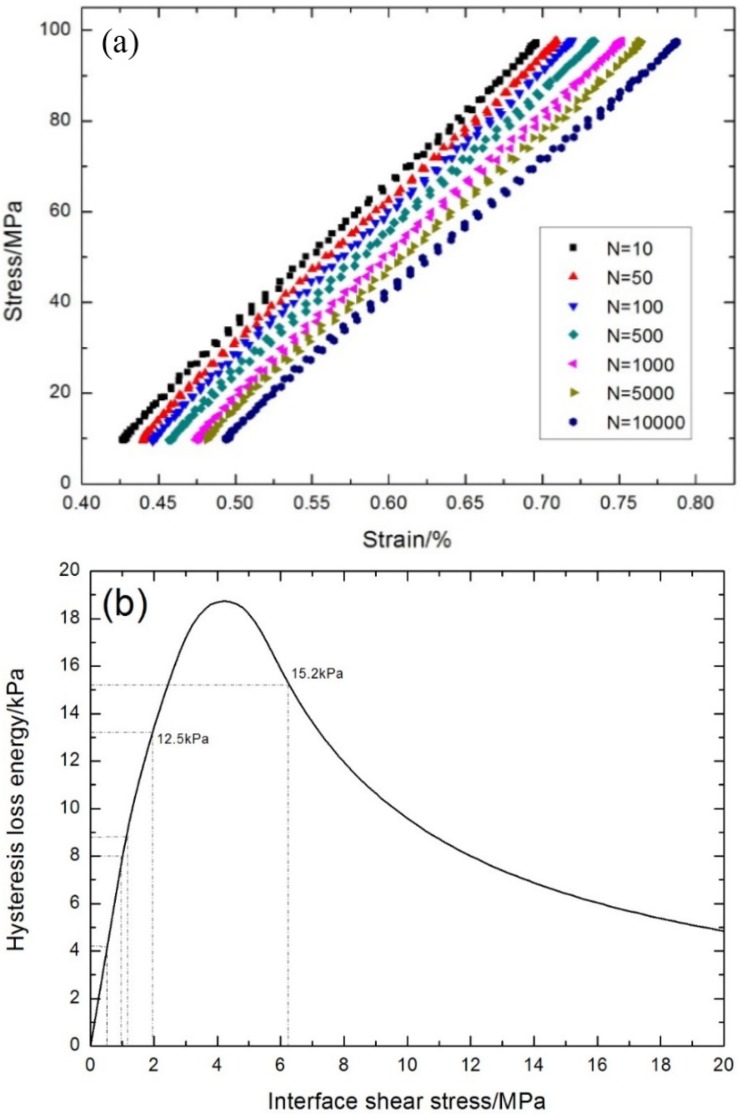
(**a**) The experimental fatigue hysteresis loops of different numbers of applied cycles; and (**b**) the theoretical fatigue hysteresis loss energy as a function of interface shear stress of cross-ply C/SiC composite under σ_max_ = 97.5 MPa at 800 °C in air.

The fatigue hysteresis loops under σ_max_ = 97.5 MPa at 800 °C in air corresponding to the 10th, 50th, 100th, 500th, 1000th, 5000th and 10,000th cycles are shown in Figure 14a. The theoretical fatigue hysteresis loss energy as a function of the interface shear stress is shown in Figure 14b. The fatigue hysteresis loss energy increases with the decreasing interface shear stress to the peak value of 18.7 kPa, corresponding to interface slip Case 1 and Case 2, *i.e.*, interface partially debonding and fiber slipping completely/partially relative to the matrix in the interface debonded region of the 0° plies; it decreases with decreasing interface shear stress to zero kPa, corresponding to interface slip Case 3 and Case 4, *i.e.*, interface completely debonding and fiber slipping partially/completely relative to the matrix in the interface debonded region of the 0° plies. Comparing experimental fatigue hysteresis loss energy with theoretical computational values, the interface shear stress corresponding to the first, second, third, fifth, 15th, 500th, 1000th, 3000th, 5000th, 10,000th and 12,000th cycles can be estimated, as shown in Table 3.

**Table 3:** The fiber/matrix interface shear stress of cross-ply C/SiC composite for different numbers of applied cycles under σ_max_ = 97.5 MPa at 800 °C in air.

Loading Cycles	Experimental Fatigue Hysteresis Loss Energy/kPa	Interface Shear Stress/MPa
1	15.2	6.2
2	12.5	2.0
5	8.8	1.1
15	8	1
500	6.6	0.85
1000	6.2	0.78
3000	5.3	0.72
5000	4.8	0.6
10,000	4.3	0.53
12,000	4.2	0.5

**Figure 15:**
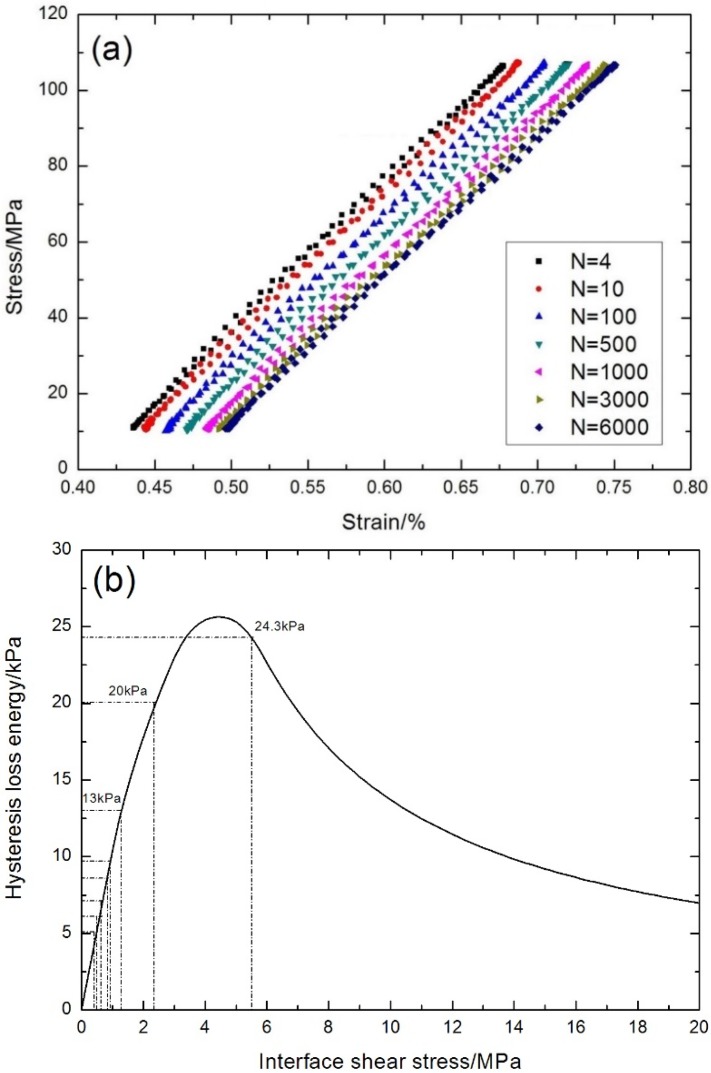
(**a**) The experimental fatigue hysteresis loops of different numbers of applied cycles; and (**b**) the theoretical fatigue hysteresis loss energy as a function of interface shear stress of cross-ply C/SiC composite under σ_max_ = 105 MPa at 800 °C in air.

The fatigue hysteresis loops under σ_max_ = 105 MPa at 800 °C in air corresponding to the fourth, 10th, 100th, 500th, 1000th, 3000th and 6000th cycles are shown in Figure 15a. The theoretical fatigue hysteresis loss energy as a function of the interface shear stress is shown in Figure 15b. The fatigue hysteresis loss energy increases with the decreasing interface shear stress to the peak value of 25.6 kPa, corresponding to interface slip Case 1 and Case 2, *i.e.*, interface partially debonding and fiber slipping completely/partially relative to the matrix in the interface debonded region of the 0° plies; it decreases with decreasing interface shear stress to zero kPa, corresponding to interface slip Case 3 and Case 4, *i.e.*, interface completely debonding and fiber slipping partially/completely relative to the matrix in the interface debonded region of the 0° plies. Comparing experimental fatigue hysteresis loss energy with theoretical computational values, the interface shear stress corresponding to the first, second, third, 10th, 100th, 500th, 1000th, 3000th and 6600th cycles can be estimated, as shown in Table 4.

**Table 4:** The fiber/matrix interface shear stress of cross-ply C/SiC composite for different numbers of applied cycles under σ_max_ = 105 MPa at 800 °C in air.

Loading Cycles	Experimental Fatigue Hysteresis Loss Energy/kPa	Interface Shear Stress/MPa
1	24.3	5.5
2	20	2.3
3	13	1.3
10	9.7	0.9
100	8.6	0.8
500	7.1	0.6
1000	6.1	0.5
3000	5.4	0.45
6600	5.1	0.4

**Figure 16:**
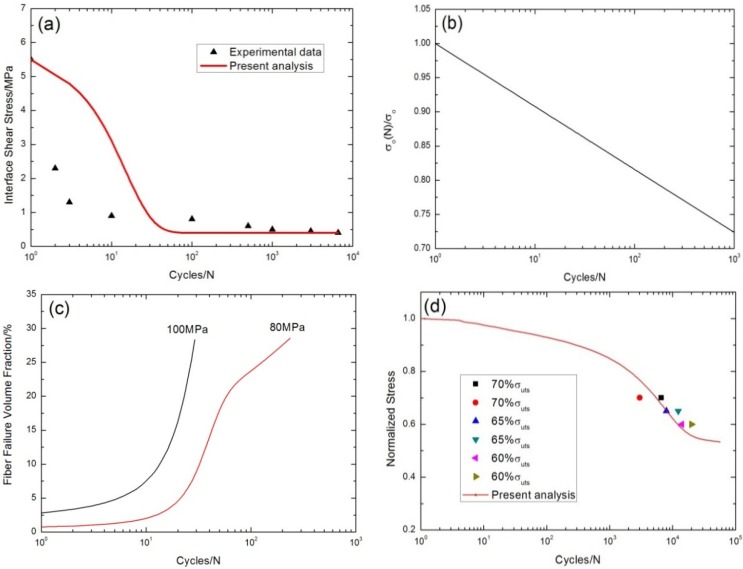
(**a**) The interface shear stress *versus* cycle number; (**b**) the fiber strength *versus* cycle number; (**c**) the fraction of broken fibers *versus* cycle number; and (**d**) the fatigue life S-N curves of experiment data and present analysis for cross-ply C/SiC composite at 800 °C in air.

The interface shear stress as a function of the cycle number has been simulated using the Evans-Zok-McMeeking model [6], as shown in Figure 16a. The model parameters are given by τ_io_ = 5.5 MPa, τ_imin_ = 0.4 MPa, ω = 0.001 and λ = 1.0. The interface shear stress degrades from 5.5 MPa at the first cycle to 0.6 MPa at the 3600th cycle due to interface oxidation, as shown in Figure 16a. The fiber strength degradation curve predicted using the Lee-Stinchcomb model [23] is shown in Figure 16b, in which *p*_1_ = 0.02 and *p*_2_ = 1. The fraction of broken fibers under σ_max_ = 100 and 80 MPa corresponding to a different cycle number predicted by the present analysis is illustrated in Figure 16c. Under σ_max_ = 80 MPa, the fraction of broken fibers *versus* the cycle number curve can be divided into two regions, *i.e.*, (1) at the initial stage of cyclic-fatigue loading, the fraction of broken fibers increases rapidly due to degradation of the interface shear stress and fiber strength; and (2) when the interface shear stress approaches the steady-state value, the fiber fracture is mainly attributed to degradation of the fiber strength, which makes the fraction of broken fibers increase slowly, as shown in Figure 16c. The experimental and predicted fatigue life S-N curves are illustrated in Figure 16d. The fatigue life at 800 °C in air is greatly reduced compared to that at room temperature, which is attributed to oxidation of PyC interphase and carbon fibers. The predicted fatigue life S-N curve can be divided into two regions, *i.e.*, (1) the A–B part is affected by the degradation of the interface shear stress and the fiber strength for interface wear and oxidation; (2) the B–C part is mainly affected by the degradation of the fiber strength for interface oxidation, as shown in Figure 16d.

## 6. Conclusions

(1) Under cyclic-fatigue loading of cross-ply C/SiC composite at room temperature, the fatigue hysteresis modulus first decreases rapidly due to transverse cracks in the 90° plies, and matrix cracks and interface debonding in the 0° plies; with increasing applied cycles, when matrix cracks approach saturation, the decrease of the fatigue hysteresis modulus would be mainly attributed to degradation of the interface shear stress for interface wear. However, at 800 °C in air, the fatigue hysteresis modulus decreases much more rapidly compared with that at room temperature.

(2) The fatigue limit of cross-ply C/SiC composite at room temperature was about 88% of the tensile strength. However, there was no apparent fatigue limit for cross-ply C/SiC composite under cyclic-fatigue loading at 800 °C in air due to the oxidation of the fiber/matrix interphase or carbon fibers.

(3) Combining the fiber fracture model with the interface shear stress degradation model and the fiber strength degradation model, the fraction of broken fibers *versus* cycle number was predicted under constant fatigue peak stress, *i.e.*, the fraction of broken fibers first increases rapidly due to degradation of the interface shear stress and the fiber strength; when the interface shear stress approaches the steady-state value, the fiber failure is mainly controlled by the degradation of the fiber strength, which makes the fraction of broken fibers increase slowly. At 800 °C in air, the degradation of the fiber strength caused by the oxidation of carbon fibers leads to apparent life reduction compared with that at room temperature.

## Nomenclature

*V*	volume fraction
α	thermal expansion coefficient
*E*	Young’s modulus
Δ*T*	temperature change from “stress-free” temperature
*l_c_*	matrix crack spacing
σ	stress
ε	strain
*y*	interface counter-slip length
*z*	interface new-slip length
*P*	broken fibers fraction
*m*	Weibull modulus
*r_f_*	fiber radius
*l_d_*	interface debonded length
τ_i_	fiber/matrix interface shear stress
τ_io_	initial interface shear stress
τ_imin_	steady-state interface shear stress
*T*	load carried by intact fibers at the matrix crack plane
*<T_b_>*	load carried by broken fibers
ε_c_unload_	unloading composite strain
ε_c_reload_	reloading composite strain
*U*	hysteresis loss energy
η	composite damage parameter
*p*_1_, *p*_2_	empirical parameter of fiber strength degradation model
ω, λ	empirical parameter of interface shear stress degradation model
Superscript and Subscript	
*f*	fiber
*m*	matrix
*c*	composite

## References

[1] Naslain R. (2004). Design, preparation and properties of non-oxide CMCs for application in engines and nuclear reactors: An overview. Compos. Sci. Technol..

[2] Schmidt S., Beyer S., Knabe H., Immich H., Meistring R., Gessler A. (2004). Advanced ceramic matrix composite materials for current and future propulsion system applications. Acta Astronaut..

[3] DiCarlo J.A., Roode M. Ceramic composite development for gas turbine hot section components. Proceedings of the GT2006 ASME Turbo Expo 2006: Power for Land, Sea and Air.

[4] Stephen T. General Electric Primes CMC for Turbine Blades. http://www.flightglobal.com/news/articles/general-electric-primes-cmc-for-turbine-blades-349834/.

[5] Zhang L.T., Cheng L.F., Luan X.G., Mei H., Xu Y.D. (2006). Environmental performance testing system for thermostructure materials appleid in aeroengines. Key Eng. Mater..

[6] Evans A.G., Zok F.W., McMeeking R.M. (1995). Fatigue of ceramic matrix composites. Acta Metall. Mater..

[7] Zhu S., Mizuno M., Kagawa Y., Mutoh Y. (1999). Monotonic tension, fatigue and creep behavior of SiC-fiber-reinforced SiC-matrix composites: A review. Compos. Sci. Technol..

[8] Mall S., Engesser J.M. (2006). Effects of frequency on fatigue behavior of CVI C/SiC at elevated temperature. Compos. Sci. Technol..

[9] Ruggles-Wrenn M.B., Christensen D.T., Chamberlain A.L., Lane J.E., Cook T.S. (2011). Effect of frequency and environment on fatigue behavior of a CVI SiC/SiC ceramic matrix composite at 1200 °C. Compos. Sci. Technol..

[10] Rouby D., Reynaud P. (1993). Fatigue behavior related to interface modification during load cycling in ceramic-matrix fiber composites. Compos. Sci. Technol..

[11] Reynaud P. (1996). Cyclic fatigue of ceramic-matrix composites at ambient and elevated temperatures. Compos. Sci. Technol..

[12] Fantozzi G., Reynaud P. (2009). Mechanical hysteresis in ceramic matrix composites. Mater. Sci. Eng. A.

[13] Kuo W.S., Chou T.W. (1995). Multiple cracking of unidirectional and cross-ply ceramic matrix composites. J. Am. Ceram. Soc..

[14] Li L.B., Song Y.D., Sun Y.C. (2014). Effect of matrix cracking on hysteresis behavior of cross-ply ceramic matrix composites. J. Compos. Mater..

[15] Li L.B. (2013). Modeling hysteresis behavior of cross-ply C/SiC ceramic matrix composites. Compos. B.

[16] Saleem M., Zitoune R., Sawi I.E., Bougherara H. (2015). Role of the surface quality on the mechanical behavior of CFRP bolted composite joints. Int. J. Fatigue.

[17] Saleem M., Toubai L., Zitoune R., Bougherara H. (2013). Investigating the effect of machining processes on the mechanical behavior of composite plates with circular holes. Compos. A.

[18] Zheng G.W. (2009). The application of PID parameters dynamic tuning method for servo fatigue testmachine. Non-Ferr. Min. Metall..

[19] Yasmin A., Bowen P. (2004). Fatigue behavior of cross-ply Nicalon/CAS II glass-ceramic matrix composite at room and elevated temperatures. Compos. A.

[20] Zhu S.J., Kaneko Y., Ochi Y., Ogasawara T., Ishikawa T. (2004). Low cycle fatigue behavior in an orthogonal three-dimensional woven Tyranno fiber reinforced Si–Ti–C–O matrix composite. Int. J. Fatigue.

[21] Curtin W.A. (2000). Stress-Strain Behavior of Brittle Matrix Composites. Comprehensive Composite Materials.

[22] Phoenix S.L., Raj R. (1992). Scalings in fracture probabilities for a brittle matrix fiber composite. Acta Metall. Mater..

[23] Lee S.S., Stinchcomb W.W. (1994). Damage mechanisms of cross-ply Nicalon/CAS-II laminate under cyclic tension. Ceram. Eng. Sci. Proc..

